# Rational Design of Optical Single-Walled Carbon Nanotube-Based Nanosensors with Biological Recognition Elements

**DOI:** 10.1002/adsr.202500076

**Published:** 2026-01-28

**Authors:** Amelia K. Ryan, Atara Israel, Maria Celina Stefoni, Catarina Ferraz, Ryan M. Williams

**Affiliations:** Department of Biomedical Engineering, The City College of New York, New York, NY 10031, USA; Department of Biomedical Engineering, The City College of New York, New York, NY 10031, USA; Department of Biomedical Engineering, The City College of New York, New York, NY 10031, USA; Departamento de Química Inorgánica, Analítica y Química Física, Facultad de Ciencias Exactas y Naturales (DQIAQF), Universidad de Buenos Aires and Instituto de Química Física de los Materiales, Medio Ambiente y Energía (INQUIMAE), CONICET-UBA, Buenos Aires C1428, Argentina; Department of Biomedical Engineering, The City College of New York, New York, NY 10031, USA; Department of Biomedical Engineering, The City College of New York, New York, NY 10031, USA; Department of Medicine, Division of Nephrology & Hypertension, Stony Brook University, Stony Brook, NY 11794, USA

**Keywords:** antibody, aptamer, molecular recognition, nanobiosensors, near-infrared fluorescence, SWCNT

## Abstract

Single-walled carbon nanotubes (SWCNT) can serve as powerful transducers for optical nanobiosensors. As near-infrared (NIR) fluorophores, they are used for a wide variety of biological sensing and imaging applications in vitro and in vivo. Rational biosensor design relies on the use of biological recognition elements to detect the sensor’s target. In rationally designing SWCNT nanobiosensors, the nanotubes are functionalized with a biological recognition motif, whose binding event induces a modulation in SWCNT fluorescence, which can be measured with NIR spectroscopy in a well plate or cuvette, in cells, or through tissue of live animals. In this review, the sensor design strategies and functional outcomes of rationally-designed optical SWCNT sensors are assessed that employ biological recognition elements for analyte specificity. The biomolecular recognition elements are divided into categories of proteins, peptides, or oligonucleotides, and assessed functionalization schemes, highlighting advances made in the fields of biomedical sensing and imaging through rational design. Finally, a perspective is offered on remaining challenges and future directions for the field of SWCNT optical sensor engineering and hurdles for translation to the clinic.

## 1. Introduction

Biosensors are analytical devices that detect a biological target, often with applications in biomedical research, environmental sensing, or clinical diagnostics. A sensor is composed of a recognition probe with selectivity for an analyte and a transducer that produces a signal to be measured by a detector. Generally, biosensors rely on biological recognition elements (e.g. antibodies, aptamers) to serve as probes to interact with the analyte.^[1]^ Biosensors facilitate scientific breakthroughs by enabling the investigation of unstudied biological processes and may also improve clinical treatments by serving as point-of-care or implantable devices capable of altering the healthcare landscape. Nanosensors, composed of an engineered nanomaterial with at least 1D less than 1,000 nm, are particularly attractive for biological applications. Due to their potential for low-cost fabrication, ultrahigh sensitivity and selectivity, biocompatibility, and real-time monitoring, nanosensors may offer beneficial alternatives to traditional diagnostic methods.^[2,3]^ Materials commonly used for nanosensor construction include polymeric nanoparticles, quantum dots, nanowires, graphene, and carbon nanotubes.^[4]^

Single-walled carbon nanotubes (SWCNT) are quasi-1D materials composed of sp^2^-hybridized carbon with an ordered electronic structure which drives unique optical properties.^[5,6]^ A large gap between the valence and conduction band in semiconducting SWCNT enables emission of near-infrared (NIR) fluorescence.^[7]^ The family of semiconducting SWCNT contains numerous species (or chiralities), each of which emits fluorescence at a distinct wavelength in the NIR region.^[8,9]^ This portion of the electromagnetic spectrum is also known as the tissue transparency window as it enables high tissue penetration depth, low background, and minimal scattering.^[10,11]^ Further, SWCNT fluorescence is innate and does not photobleach, offering a significant advantage over traditionally-used organic fluorophores.^[12]^

## 2. SWCNT Functionalization Schemes

Preparation of SWCNT optical sensors requires a few key steps: first, nanotubes must be suspended and isolated in an aqueous solution. SWCNT are inherently hydrophobic and therefore prone to aggregation, which quenches fluorescence.^[7]^ Probe-tip sonication of SWCNT with a suitable dispersant serves to colloidally stabilize single nanotubes, allowing them to fluoresce and deploy readily in biological systems. Bile salt surfactants, pluronics, and other polymers are commonly used.^[13–19]^ Additionally, biological molecules have been used to solubilize SWCNT, including proteins, lipids, and peptides.^[20–24]^ Oligonucleotides are frequently utilized for SWCNT dispersion as well, wherein ring structures in each base form strong *π*-stacking interactions on the SWCNT surface and the phosphate backbone provides electrostatic repulsion for stable aqueous solutions.^[25–30]^ Single-stranded DNA and RNA are highly versatile, with a virtually unlimited library of sequences and vast potential for chemical modifications, in addition to being commercially available at low-cost.

Though semiconducting SWCNT fluorescence is intrinsic and modifiable, these properties are not specific toward any analyte without further engineering of the nanotube. SWCNT can be functionalized with biorecognition elements to target the desired biomarker. Careful selection of the biorecognition motif is critical for effective biosensor design – proteins, peptides, and oligonucleotides which are complementary to the target analyte have been used for developing SWCNT sensors.^[31–33]^ The native binding activity of these recognition motifs with their target enable high sensitivity, specificity, and selectivity of SWCNT optical sensors, particularly in complex biological environments.^[4,34]^ Such approaches are referred to as rational design, as they select a binding element a priori to sensor synthesis, as opposed to screening-based or computational approaches.

Noncovalent functionalization methods maintain the native optical properties of SWCNT and do not require specialized chemistry.^[21,35–37]^ In some cases, the dispersant and recognition element are one and the same. SWCNT can be directly sonicated with the recognition probe – a method often employed with oligonucleotide probes which can effectively solubilize, isolate, and functionalize SWCNT simultaneously.^[25,36,38]^ While the direct sonication approach offers high efficiency with minimal steps, the conformation adopted on the SWCNT surface may not be optimal for binding to a target analyte. Some studies using oligonucleotide binding elements perform additional modifications on the nanotube surface, such as ionically and thermally induced denaturation and refolding of the oligonucleotide to achieve an ideal conformation for analyte binding.^[39]^ Another approach to noncovalent functionalization is sonication of SWCNT with a polymer or surfactant followed by dialysis to exchange the surface layer to the biorecognition motif of choice.^[40]^ The dialysis procedure can be time-consuming, but this technique preserves the function and structure of the chosen protein while coating it on the nanotube surface. This method has been used with proteins whose structures may be disrupted by the direct sonication process. Finally, SWCNT can be sonicated with an intermediate linker such as a (bio)polymer with a chemical modification which will allow for later conjugation of the biorecognition probe.^[19,41–44]^ This technique has been employed with antibodies, peptides, and other proteins and offers stable conjugates while maintaining the native properties of both the SWCNT and the binding motif, though it does involve extra time-consuming steps to complete the conjugation.

Covalent functionalization approaches introduce new bonds to the SWCNT carbon lattice which leads to highly stable conjugates.^[45,46]^ While uncontrolled addition of sp^3^ defects disrupt the graphitic structure and diminish fluorescence, recent advances controlled the chemistry and stoichiometry of the reaction to develop SWCNT with synthetic quantum defects, or organic color centers.^[47,48]^ Organic color centers create an additional bright photoluminescence peak and offer opportunities for addition of DNA, antibody fragments, or other biological recognition elements for sensing applications.^[32,49]^

Beyond rationally designed sensors with biorecognition elements, screening-based approaches are regularly used to design SWCNT sensors. Screening approaches use a shotgun approach, with a library of polymer-wrapped SWCNT, to determine which has the highest affinity and selectivity for various analytes. The polymers used in this method form a molecular recognition corona on the SWCNT surface which causes a modulation in nanotube fluorescence upon analyte binding. The Corona Phase Molecular Recognition (CoPhMoRe) approach has been utilized to detect a wide variety of biologically relevant targets.^[8,50–56]^ While this review will focus on applications of known biorecognition elements, the widely-used and strongly successful screening-based approaches have been reviewed extensively elsewhere.^[35,57–59]^

## 3. Signal Transduction

In the context of SWCNT optical biosensors, signal transduction is the conversion of a molecular recognition event into a measurable change in nanotube fluorescence, typically in the form of intensity changes and/or wavelength shifts. The signal transduction pathway is dependent upon the nature of the recognition element, its attachment to the SWCNT, and the dynamics of SWCNT excitons.^[33,60]^

**Figure 1.**
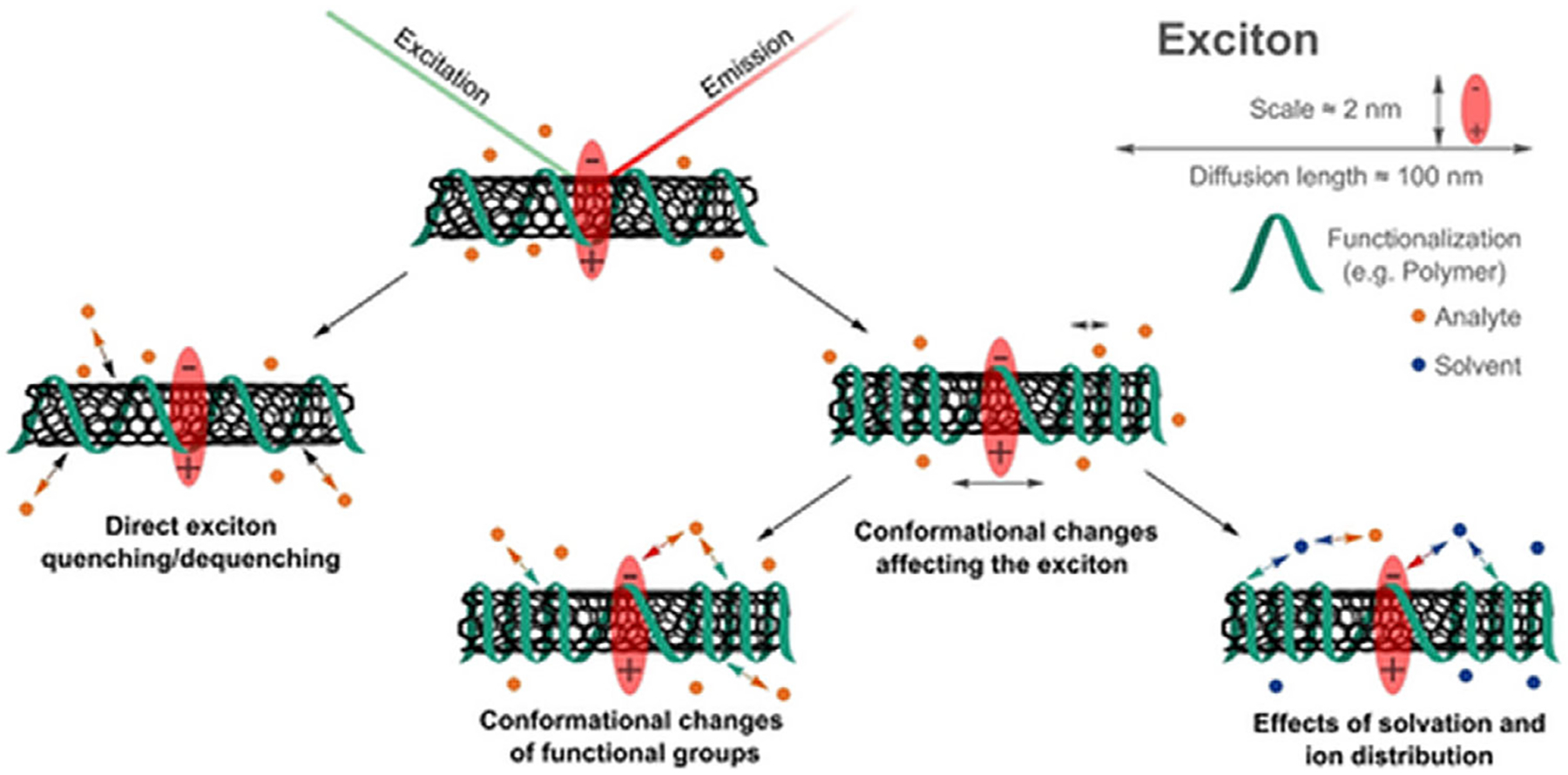
Mechanisms of fluorescence modulation in SWCNT sensors. Reprinted Open Access Ackermann et al. Angewandte Chemie International Edition 2022^4^.

Though not often observed in rationally designed sensors, some SWCNT constructs exhibit direct fluorescence quenching through adsorption of the analyte to the SWCNT surface rather than interacting with a biorecognition element. Rational sensor design typically entails direct analyte binding which alters the hydration, packing density, or conformation of the corona phase (Figure 1). This induces local changes in dielectric constant near the SWCNT surface, which shift exciton energy, causing red- or blue-shifts.^[42,61]^ Displacement of water or DNA from the SWCNT surface generally leads to blue-shifts and increase in fluorescence intensity, while red-shifting and fluorescence quenching can be caused by DNA binding to the nanotube surface and local charge increase.^[41,62–64]^

The choice of biorecognition element will directly influence the signal transduction method. The orientation and surface coverage of the recognition element on the SWCNT, as well as its structural flexibility, determine conformational alterations in the corona. Further, the method in which the recognition element is attached to the SWCNT can also influence signal transduction method. Covalently and noncovalently functionalized sensors differ in their susceptibility to exciton quenching, interference in physiological conditions, and signal-to-noise ratio.^[35,46]^

**Figure 2.**
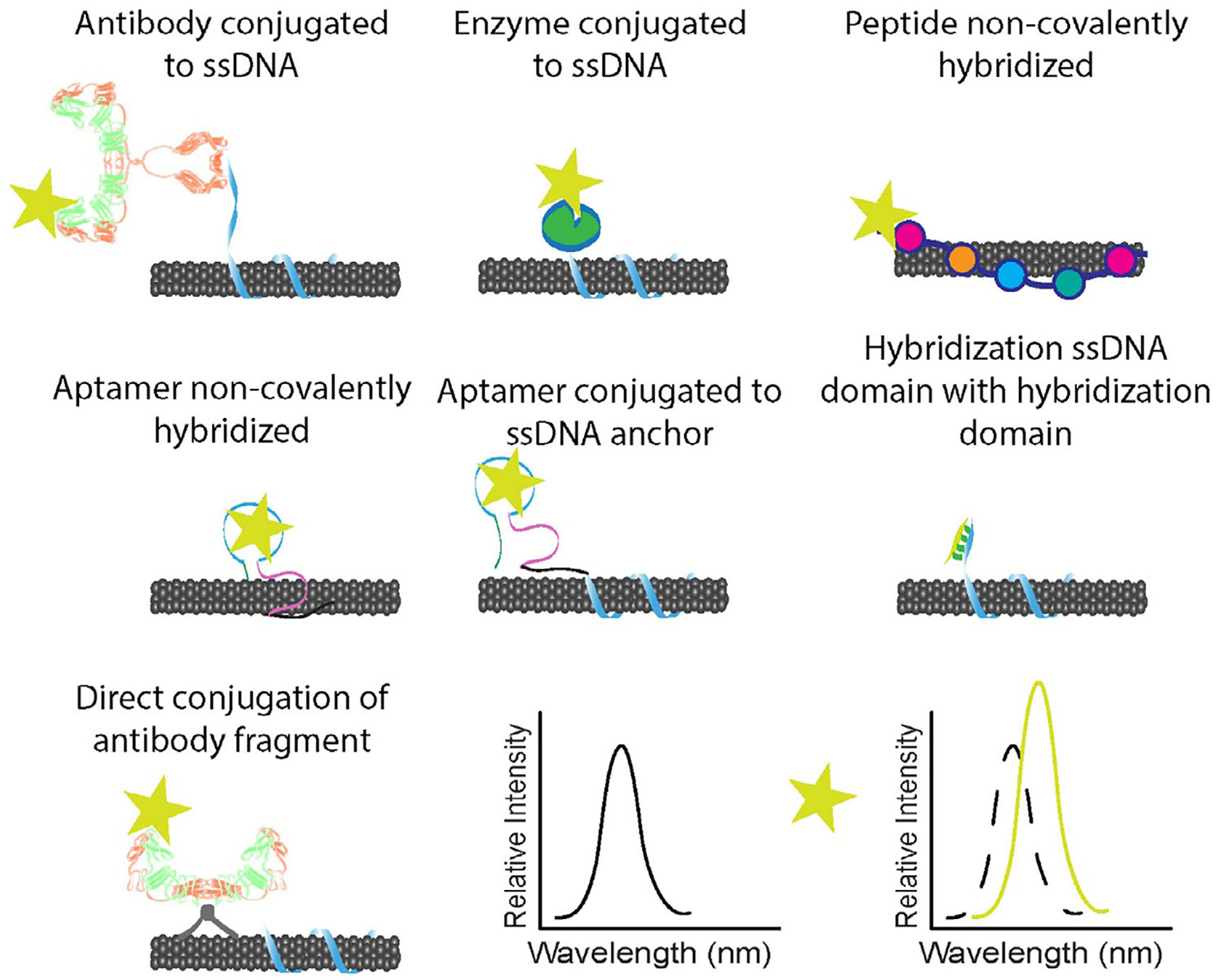
Overview of strategies to rationally design SWCNT based sensors with biomolecular recognition elements and near-infrared sensing example.

In this review, we focused on assessing functionalization schemes and performance outcomes of SWCNT transducers coupled with biomolecular recognition elements, categorized proteins, peptides, and oligonucleotides, as well as methods of signal transduction. We also assessed various strategies used to optimize the innate function of the biorecognition motifs while simultaneously engineering an effective nanosensor (Figure 2). Finally, we offered our perspective on the remaining challenges and future directions for the field.

## 4. Proteins as Biorecognition Elements

Proteins are among the most versatile biorecognition elements for SWCNT-based sensors, offering high binding specificities toward a wide range of biological and chemical targets (Table 1).^[65,66]^ Their structural complexity and diversity of functional groups enable selective analyte recognition through well-defined active sites, often with sub-nanomolar affinities. In SWCNT-based sensing, proteins can be immobilized noncovalently or covalently onto the nanotube surface, where binding events can trigger detectable changes in fluorescence intensity or wavelength. However, the inherent size, structural sensitivity, and potential instability of proteins require careful attachment strategies to preserve activity while maintaining contact with the SWCNT surface for efficient signal generation.

### 4.1. Antibodies and Antibody Fragments

Antibodies are widely utilized as biomolecular recognition elements because of their high affinity toward targets of interest, allowing for highly specific and selective biosensors.^[67–69]^ The first instance of an optical antibody-based SWCNT sensor was designed to target human cardiac troponin T (cTnT), a cardiac regulatory protein that is released into the bloodstream at the onset of acute myocardial infarction.^[70]^ SWCNT were suspended with chitosan and crosslinked onto a poly-L-lysine (PLK)-coated glass slide, resulting in a thin immobilized gel. Nickel-NTA chemistry was used to bind cTnT antibodies. The sensor demonstrated a limit of detection (LOD) of 100 ng mL^−1^ cTnT in buffer and 7 μg mL^−1^ in human plasma.

Solution-phase antibody sensors were subsequently designed using ssDNA-wrapped SWCNT. The ssDNA incorporated a primary amine, to which direct antibody conjugation was performed via carbodiimide chemistry (Figure 2, top left). This was initially developed with an antibody to detect urokinase plasminogen activator (uPA), a prostate cancer biomarker.^[41]^ The antibody-conjugated sensor exhibited an LOD of 100 pm uPA in buffer and 25 nm in 10% fetal bovine serum (FBS). In another study using the same crosslinking method, a nanosensor was engineered for human epididymis protein-4 (HE4) – an ovarian cancer biomarker with the potential to help clinicians differentiate between benign and malignant ovarian lesions.^[42]^ The sensor demonstrated sensitivity to HE4 with an LOD of 10 nm when tested in 10% FBS. A glass chip-based sensor was constructed to detect HE4 in patient ascites and serum samples using as little as 10 μL of patient fluid. Additionally, the SWCNT-anti-HE4 sensor was encapsulated in a semi-permeable dialysis membrane device and surgically implanted into live mice, successfully detecting HE4 produced by intraperitoneally-implanted human cell lines compared to two HE4-null lines. This is the first work that demonstrated in vivo molecular sensing using antibody-conjugated SWCNT, a significant advancement in the field.

SWCNT surface passivation is a useful tool for implementation in complex biological environments as it aims to inhibit nonspecific protein interactions. In the uPA and HE4 sensor papers above, bovine serum albumin (BSA) was used as a passivation agent to prevent non-specific interference. Subsequently, BSA and a small library of rationally-chosen passivation agents were screened using an interleukin-6 (IL-6)-antibody-conjugated sensor.^[71]^ In the absence of passivation, a nanosensor’s fluorescence in FBS exhibited red-shifting of most chiralities due to nonspecific serum proteins adsorbing to the surface of the nanotube. PLK and BSA were identified as excellent passivation agents for the antibody-conjugated sensor – both enabled an LOD of 25 pg mL^−1^ IL-6 in human serum.

An anti-estrogen receptor alpha (ER*α*) antibody conjugated to SWCNT was used to differentiate between tumors with or without estrogen receptor expression in breast cancer patient samples.^[44]^ While previous antibody-based SWCNT sensors had been used to detect soluble proteins, this sensor was the first to detect a biomarker on the plasma membrane of a cell. The sensor was cross-referenced against standard immunohistochemistry, demonstrating a 94% accuracy in differentiating between ER− and ER+ samples.

While not a quantitative molecular sensor, a SWCNT-antibody probe for detection and imaging of breast cancer cells has also been designed. SWCNT were suspended with phospholipid-polyethylene glycol amine (PL-PEG-NH_2_), which served as an intermediate linker and an inhibitor of nonspecific binding.^[72]^ The nanotubes were then separately conjugated with two monoclonal antibodies used therapeutically in the clinic: Rituxan (anti-CD20) and Herceptin (anti-HER2/neu). Both imaging probes demonstrated selective binding to cancer cells expressing their target analyte, indicated by NIR fluorescence measurements.

M13 bacteriophages have also been implemented as an intermediate to functionalize antibodies onto SWCNT. M13 bacteriophages serve as a versatile scaffold for functionalization due to its five capsid proteins which can be genetically engineered to display targeting motifs. This construct was used to create a targeted imaging probe for tumors.^[73,74]^ The M13 scaffold was engineered to express both a SWCNT binding peptide and a peptide handle for later site-specific conjugation of an anti-prostate specific membrane antigen (PSMA) antibody. Again, while not a molecular sensor, the imaging agent showed 8.3-folder higher uptake into PSMA-positive human prostate cancer cells compared to PSMA-negative cells. The SWCNT-M13 platform has also been used as a bacterial probe to discriminate between *F’*-positive and *F’*-negative strains of *Staphylococcus aureus (S. aureus)*.^[75]^ The anti-*S. aureus* antibody was conjugated to the SWCNT-M13 platform using a streptavidin-biotin reaction. After injecting mice with an *S. aureus* strain, both the antibody-functionalized SWCNT-M13 and generic SWCNT-M13 were deployed in vivo. Results showed a 3.4x fluorescence intensity enhancement from SWCNT-anti-*S. aureus*-M13 in detection of intramuscular infections and 5.7x enhancement in imaging infective endocarditis.

Nanobodies are small antibody fragments derived from camelids with a unique tertiary structure consisting of only a single variable domain.^[76,77]^ Nanobodies were used in conjunction with SWCNT to image kinesin-5-green fluorescent protein (GFP)-labeled motor proteins in *Drosophila melanogaster* embryos.^[78]^ ssDNA-encapsulated SWCNT were conjugated to a GFP-binding nanobody using maleimide chemistry. The probe was then deployed for deep tissue imaging in *Drosophila* embryos to track the expression and movement of motor protein kinesin-5 as a GFP-fusion protein. The nanobody-GFP binding event allowed SWCNT fluorescence to be easily detected in the embryos. The ability to track Kinesin-5 movement is beneficial for monitoring the development of *Drosophila*. This was the first instance of SWCNT-nanobody conjugates. The same GFP-binding nanobody was later covalently conjugated to (*N*-maleimido)phenyl (MalPh) quantum defect-SWCNT.^[46]^ The sensor was immobilized on a glass substrate and incubated with GFP. Colocalization of GFP and NIR fluorescence confirmed the functionality of the sensor, marking the first covalent conjugation of a functional protein to a SWCNT with preserved NIR fluorescence.

Another innovative example is the use of a supercharged single-chain antibody fragment (scFv) coupled with covalent SWCNT functionalization for the detection of human IL-6.^[79]^ The engineered scFv underwent an irreversible conformational change upon binding to IL-6. The scFv was transformed to a permanent folding state and conjugated to the SWCNT surface with a 4-carboxyl aryl defect via carbodiimide chemistry (Figure 2, bottom left). A robust shift of the defect fluorescence emission was observed upon IL-6 binding with an LOD of 80 nm. The scFv yielded superior sensing ability over non-supercharged antibody fragments: the magnitude of the response was greater for the supercharged platform, attributed to a larger conformational change and charge transfer.

### 4.2. Enzymes

Among the first-used biological recognition elements employed for SWCNT optical sensing was glucose oxidase (GOX), an enzyme that catalyzes the reaction of *β*-D-glucose to the D-glucono-1,5-lactone with a H_2_O_2_ co-product.^[10,11]^ SWCNT were sonicated and dispersed in a sodium cholate solution, followed by dialysis in the presence of GOX. As a result, the surfactant was replaced with an immobilized, porous layer of protein on the SWCNT surface. Then, redox mediator potassium ferricyanide, K_3_Fe(CN)_6_, was irreversibly adsorbed to the SWCNT surface to quench fluorescence emission through a partial electron transfer. When glucose was oxidized, the H_2_O_2_ that appeared in solution reduced the K_3_Fe(CN)_6_ and reversed the electron transfer, restoring SWCNT emission in a turn-on format. The sensor functioned within the general range of blood glucose in diabetic patients, with an LOD of 34.7 μm.

Additional approaches have also been used to develop SWCNT-GOX optical sensors. One study dispersed SWCNT with ssDNA before immobilizing GOX and adding K_3_Fe(CN)_6_ as a redox mediator.^[80]^ While in the previous work GOX coated directly on the SWCNT surface had lost some of its enzymatic activity, the addition of a DNA interface between the nanotube and enzyme allowed GOX to maintain its native function. Another study used SWCNT-GOX without the addition of K_3_Fe(CN)_6_ as an electroactive species,^[81]^ allowing GOX to act as an electron donor to p-doped sites on the nanotube – which themselves were induced by oxygen on SWCNT defect sites. The oxygenated p-doped sites trapped excitons to decrease fluorescence emission.^[82]^ As glucose reacted with GOX, the extracted charge passivated the oxygenated site, yielding an increase in fluorescence intensity.^[83]^ The mechanism of SWCNT-GOX sensors was further investigated in a more recent study, which found that glucose binding to GOX itself is the dominant cause of fluorescence modulation. This new finding lead to the use of apo-GOX, a catalytically inactive form of GOX, as a recognition element for a quantitative and reversible glucose sensor with an LOD of 42 μm. Using an inactive enzyme may be advantageous for sensing applications, as enzyme activity can compromise the reversibility of sensors and consume the analyte during measurements.^[84]^

Luciferase, an oxidative enzyme that produces bioluminescence, is often used to quantify adenosine triphosphate (ATP). However, luciferase assays are time-consuming and prone to low signal-to-noise ratios. A SWCNT-Luciferase sensor was engineered to circumvent these limitations.^[85]^ SWCNT were suspended with phospholipids bearing carboxylated poly(ethylene glycol) (PLPEG-COOH). The carboxylic acid was then used to attach firefly luciferase via carbodiimide crosslinking. The sensor responded rapidly with high specificity toward ATP in solution (with an LOD of 240 nm) and in live HeLa cells.

**Figure 3.**
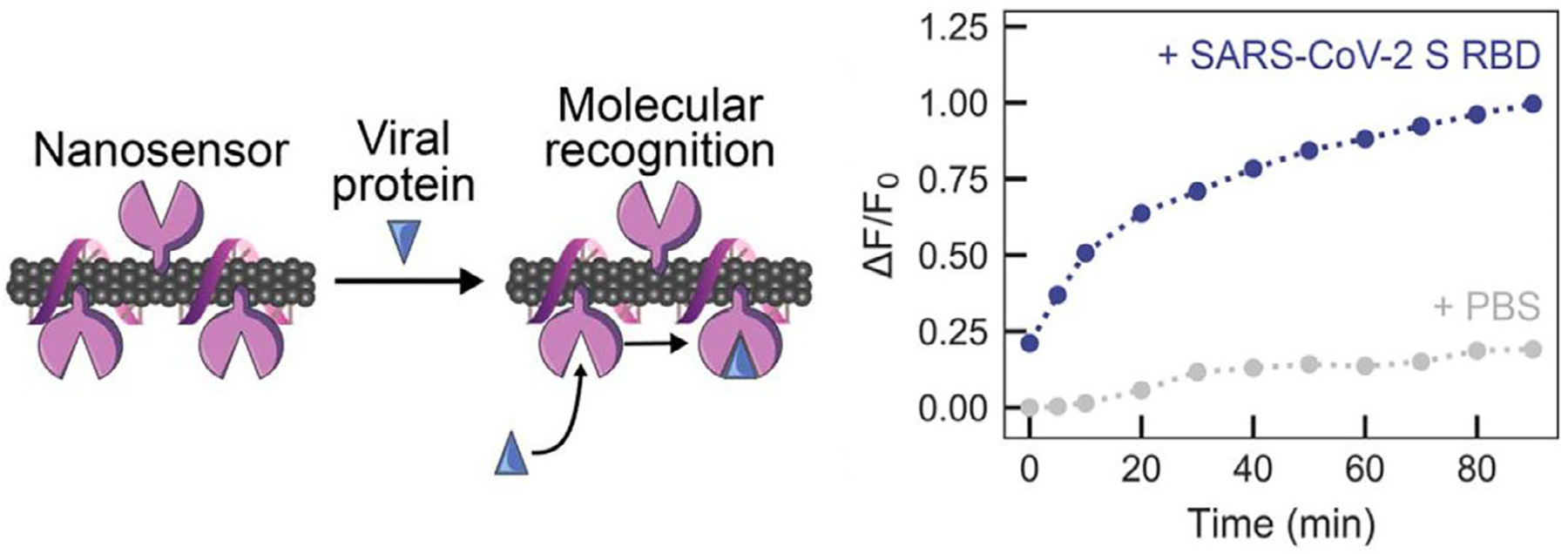
SWCNT fluorescence intensity increases upon molecular recognition of SARS-CoV-2 spike protein. (Reprinted with permission from the American Chemical Society from Pinals *et al. Nano Letters* 2021.^[86]^

Another SWCNT-enzyme sensor was designed to detect the spike protein of SARS-CoV-2.^[86]^ SWCNT-ssDNA was coated with angiotensin-converting enzyme 2 (ACE2) as a sensing protein (Figure 2, top center). ACE2 adsorption quenched SWCNT fluorescence, while binding of the Cov-2 spike protein receptor binding domain to ACE2 caused an increase in SWCNT fluorescence intensity (Figure 3). The surface-immobilized sensor responded to virus-like particles with spike protein with detection as low as ≈10^4^ viral copies per μL, matching the upper range of a patient’s viral load. The sensor displayed higher binding affinity to SARS-CoV-2 in comparison to other spike proteins as demonstrated by faster binding kinetics.

Covalent functionalization has also been used to attach enzymes to SWCNT. Azide-based triazine chemistry enabled covalent attachment of horseradish peroxidase to SWCNT, creating a hydrogen peroxide sensor with an LOD of 31 μM.^[87]^ The synthesis strategy developed in this study offered higher sensor stability and retention of native enzyme function compared to noncovalent adsorption of the same enzyme to SWCNT.^[88]^

Though outside the scope of this review, other strategies have been employed to monitor enzyme activity with SWCNT, such as substrate adsorption to SWCNT or sensing of enzymatic by-products.^[89]^

### 4.3. Additional Binding Proteins

While antibodies and enzymes have been used for their high analyte specificity, other selective protein binding interactions have been exploited as well. The first label-free sensing platform using SWCNT fluorescence to detect analyte-protein interactions used SWCNT suspended in chitosan which was chemically modified to display chelated nickel groups.^[90]^ The nickel groups acted as a docking site for a His-tagged capture protein and a proximity quencher of SWCNT emission. When anti-His-tag antibody was added to the sensor array, the distance between nickel and SWCNT increased, causing an increase in nanotube fluorescence intensity. This detection scheme was used to probe multiple protein-protein interactions, with analyte proteins such as p16, CDK4, FOS, JUN, and IgG.^[90,91]^

Protein A was used as a biomolecular recognition motif to capture IgG antibodies. This exploited the same methodology as the His-tag method above, using copper instead of nickel as a proximity quencher.^[92]^ The sensor showed enhanced affinity toward IgG with a positive correlation between intensity and IgG concentrations, achieving a LOD of 10 ng mL^−1^. The SWCNT sensing system was further developed toward multiplexing, integrating proteins A, G and L for the detection of immunoglobulins IgG, IgM, IgG2a, and IgD.^[93]^ SWCNT were noncovalently functionalized with the immunoglobulin-binding proteins and printed onto a microarray. Upon exposure to IgG, there was an increase in relative SWCNT intensity, demonstrating a linear response with increasing concentrations and an LOD of 25 μg mL^−1^. The arrays were also evaluated for their specificity and selectivity toward different immunoglobulins, which was confirmed based on the binding kinetics of the immunoglobulins tested against each of the capture proteins. As an example, protein L showed faster binding kinetics toward IgM compared to protein A or G, while IgG2a showed faster binding, and therefore higher affinity toward protein A. While there had been previous examples of SWCNT for multiplexed optical sensing, this study was the first to achieve it using different specific binding proteins for their respective targets.

Another study utilized His-tagged proteins tethered to nickel complexes for glycan profiling.^[19]^ Two high-affinity lectin-glycan pairs detected: fucose to PA-IIL lectin and *N*-acetylglucosamine to GafD lectin. In addition to analyte detection, this study identified the location of strong transducers within a SWCNT array, which interestingly were not the brightest nanotubes. This advancement assisted the future development of nanoarray sensing platforms made using SWCNT transducers.

One study sought to use methylene blue (MB) as a fluorescence resonance energy transfer-based modulator of SWCNT fluorescence.^[94]^ DNA conjugated to MB as a proximity quencher and biotin as a specific binding protein was used to suspend SWCNT. The resulting nanosensors exhibited a strong turn-on optical response to avidin, due to its high binding affinity to biotin. The study highlighted the potential of MB-tagging for other biorecognition elements (such as aptamers or antibodies) to enable robust and specific SWCNT biosensing.

A sensor for the detection of glucose was developed using glucose binding protein (GBP), a type of periplasmic binding protein that undergoes a conformational change upon analyte binding.^[95]^ GBP was conjugated to polyvinyl alcohol on the SWCNT surface. Glucose binding to GBP elicited SWCNT fluorescence quenching at concentrations from 2.5–50 mm of glucose. The sensor also demonstrated signal reversibility upon the removal of glucose from the SWCNT surface. Another SWCNT sensor targeted glucose using Concanavalin A (ConA), a plant lectin that contains polysaccharide binding sites.^[96]^ SWCNT suspended with phenoxy-derivatized dextran demonstrated aggregation and fluorescence quenching with increasing concentrations of ConA. The subsequent addition of glucose disrupted the aggregates and recovered fluorescence because of competitive binding between glucose and dextran on ConA.

Another imaging probe was created using a HaloTag protein, conjugated to SWCNT suspended with ssDNA to enable covalent linkage to full length kinesins to study cell movement and dynamics.^[97]^ When deployed in mammalian cells, the sensor targeted the kinesin-1-motor protein. Recordings of single molecule tracking showed both ordered and random movement of SWCNT, confirming that the kinase proteins were linked to the microtubule. While not a quantitative sensor, this application of SWCNT offers longer cell tracking times than traditional fluorophores due to high photostability.

### 4.4. Summary of Protein-Based Approaches

Across reported SWCNT sensors, protein functionalization strategies span noncovalent adsorption, covalent conjugation, and affinity tag-mediated immobilization, each offering a different balance between stability and retention of biological activity. Signal transduction most often occurs through fluorescence intensity changes or solvatochromic shifts, driven by conformational rearrangements and local dielectric modulation upon analyte binding. Proteins excel when high specificity is needed for native ligands, but they can be prone to denaturation, limited shelf-life, and slower response kinetics due to their size. Successful designs consistently minimize structural perturbation during immobilization and orient binding interfaces to maximize coupling with the SWCNT optical field. Going forward, site-specific conjugation and the integration of catalytic proteins near defect-modified SWCNTs could further enhance sensitivity and enable robust ratiometric readouts.

## 5. Peptides as Biorecognition Elements

Peptides are short amino acid chains linked by peptide bonds. They provide a modular and tunable alternative to full-length proteins in SWCNT sensing, combining ease of synthesis with the ability to engineer specific binding motifs. (Table 2).^[99]^ Their shorter length and simplified structures reduce steric hindrance, often allowing faster analyte diffusion to the recognition site and improved stability. In SWCNT-based sensors, peptides typically adsorb to the nanotube via aromatic and hydrophobic residues, while functional side chains interact selectively with the target analyte. This dual-interface design allows peptide binding events to directly perturb the local dielectric environment or facilitate charge transfer to the SWCNT, producing measurable optical modulation. Because peptide sequence, length, and charge distribution can be rationally tuned, they are particularly attractive for systematic structure–function studies and for designing recognition layers that balance binding specificity with robust SWCNT dispersion. Common approaches to peptide design are the use of computational simulations or shortening target-binding proteins.^[100,101]^ Peptide chemistry has enabled efficient and targeted molecular imaging through PET/SPECT, MRI, photoacoustic, and optical moieties.^[102,103]^

**Figure 4.**
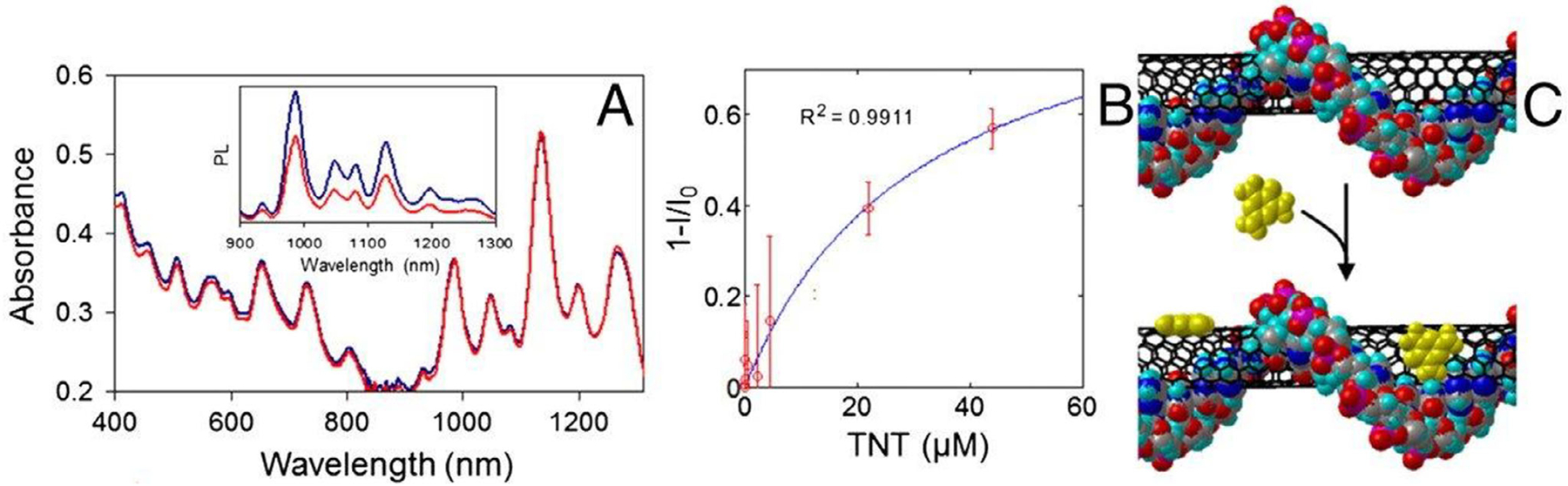
Detection of TNT using a peptide chaperone sensor. A) Sensor absorbance and photoluminescence quention upon TNT binding (inset), B) Intensity response as a function of TNT addition. C) Molecular dynamics simulation showing TNT interaction with the peptide and SWCNT surface. Copyright 2011 National Academy of Sciences, Heller et al. *PNAS* 2011.^[22]^

The first example of a SWCNT-peptide optical sensor utilized the peptide bombolitin II to detect nitroaromatics (Figure 4).^[22]^ This work introduced the concept of a chaperone sensor, wherein peptide-dispersed SWCNT indirectly detected the binding event by transducing conformational changes of the peptide upon analyte recognition (Figure 2, top right). The sensor responded to a variety of nitroaromatic compounds, with wavelength shifts that enabled fingerprinting to distinguish between analytes. This nanosensor was later implemented for detection of picric acid in live plants, technology which allowed plants to act as chemical monitors of groundwater.^[104]^

Alternatively, peptides can be conjugated to ssDNA adsorbed to SWCNT. An integrin-binding peptide sequence was conjugated to ssDNA-NH_2_ adsorbed to SWCNT using succinimidyl 4-(N-maleimidomethyl)cyclohexane-1-carboxylate (SMCC) as a crosslinker for bioconjugation to *α*_IIb_*β*_3_ integrin for a targeted NIR imaging probe.^[105]^ The binding affinities and conformational freedom of the peptide structures were tuned by their confinement to the SWCNT. Key elements used to modulate integrin affinity were DNA sequence, DNA length and geometry of conjugation. This study demonstrated the potential of peptides as a modular recognition element for SWCNT-facilitated bioimaging or cell targeting.

Building on simpler adsorption methods, a peptide-based SWCNT sensor was designed for the Alzheimer’s disease marker amyloid-beta (A*β*), which has a strong propensity for self-aggregation.^[106]^ Hydrophobic interactions and *π-π* bonds between A*β*_42_ peptide and SWCNT enabled nanotube dispersion while maintaining peptide properties. This sensor demonstrated SWCNT solvatochromism with an LOD of 100 nM in buffer conditions, disaggregation of A*β* fibrils in cell models, and intracranial detection in live mice.

A lipopolysaccharide (LPS)-binding peptide was used as part of a multiplexed SWCNT sensor platform which aimed to remotely identify clinically relevant bacteria.^[107]^ The peptide was conjugated to SWCNT-ssDNA using SMCC as a crosslinker. Upon binding to LPS, SWCNT fluorescence increased in a concentration-dependent manner in the presence of *E. coli* LPS, with a *K*_d_ value of 1.87 μM. LPS from other bacteria such as *Salmonella spp., P. aeruginosa and K. pneumoniae*, demonstrated smaller fluorescence responses.

The tripeptide glutathione has also been utilized as a biorecognition element for its affinity to bind glutathione s-transferase (GST) proteins.^[108]^ Glutathione was conjugated to acrydite-functionalized ssDNA which was then used to suspend SWCNT. The sensor responded with specificity to both GST and GST-fusion proteins, exhibiting a LOD of 1 nM GST. This study was unique as the first instance of SWCNT sensing using a small molecule as the protein recognition element.

### 5.1. Summary of Peptide-Based Sensors

Peptide-based SWCNT sensors leverage the modularity of short sequences to create recognition layers with tunable affinity, stability, and optical response. Functionalization of the SWCNT typically occurs via direct adsorption through aromatic and hydrophobic residues, or through hybrid peptide–polymer/DNA coronas for enhanced dispersion and selectivity. Signal transduction often arises from analyte-induced folding or unfolding that alters the local dielectric, or from charge transfer facilitated by strategically-placed charged or redox-active residues. Peptides offer chemical stability, rapid response times, and rational design flexibility, though their specificity is often lower than that of full proteins and their structural stability can be context-dependent. Design trends favor sequences that balance strong SWCNT adsorption with target recognition, and that incorporate aromatic residues to anchor the corona. Coupling peptide engineering with computational sequence screening could accelerate discovery of high-performance peptide–SWCNT sensors.

## 6. Oligonucleotides as Biorecognition Elements

Oligonucleotides have been well verified as an easy and effective way to disperse and isolate carbon nanotubes by sonication (Table 3).^[25,26,36,38]^ Molecular modeling studies have shown that carbon nanotubes and ssDNA interact through *π-π* stacking between DNA bases and the nanotube surface, as well as non-Watson-Crick hydrogen bonds.^[30]^ SWCNT-DNA interactions lead to water exclusion from the nanotube surface – since water quenches SWCNT fluorescence, its exclusion results in increased fluorescence.^[28]^ Furthermore, solubilization with oligonucleotides imparts biocompatibility to SWCNT.^[109]^ Long-term in vivo studies have shown no significant impacts in tissue histology, mouse weight or serum biomarkers (blood count, renal biomarkers, and hepatic markers) due to the presence of SWCNT-DNA. Due to their chemical stability, ease of synthesis, and tunability, oligonucleotides are widely-used recognition elements in SWCNT optical sensing, though their performance can be sensitive to ionic strength, temperature, and the complexity of the sensing environment.

### 6.1. Aptamers

Aptamers are short, synthetic nucleic acid sequences that are selected in vitro to specifically bind to a target molecule with high affinity. They are utilized for their excellent stability, small size, ease of chemical manipulation, and reproducible synthesis.^[110,111]^ Aptamers have been employed as recognition elements for SWCNT transducers due to their high analyte affinity in addition to the previously mentioned SWCNT-DNA interactions.

Some approaches to SWCNT-DNA sensors utilize bifunctional DNA sequences, with one encapsulating the SWCNT surface and another region binding to the analyte (Figure 2, middle center). A sensor array was designed using this method to selectively detect proteins from *E. Coli* (bacteria) and *Pichia pastoris* (yeast) at the single-molecule level.^[39]^ Custom oligonucleotides were sonicated with SWCNT and the study found that the addition of an anchor domain to the aptamer improved its functionality as a recognition element. The study also explored the impact of hexaethyleneglycol spacers to separate the aptamer and anchor domains of the polynucleotide. The presence of spacers further improved sensor response, potentially because the oligonucleotide with spacers favors the aptamer’s folded G-quadruplex structure, enhancing its binding affinity. An insulin sensor was also engineered using an aptamer-anchor oligonucleotide adsorbed to SWCNT.^[112]^ In that study, SWCNT were suspended with sodium cholate, which was later exchanged for the oligonucleotide to avoid conformational disruption of the aptamer during sonication. The aptamer enabled sensitive detection of insulin, with an LOD of 0.13 μg mL^−1^. Another study attached a platelet-derived growth factor (PDGF)-binding aptamer to SWCNT with the same exchange method, then further explored modifications of the SWCNT-aptamer construct.^[113]^ The aptamer was thermally and ionically denatured and allowed to refold on the surface of the SWCNT. After refolding, the nanosensor exhibited improved sensitivity and concentration-dependent response across 0.1–10 nM PDGF.

Several other studies have used unmodified aptamers to disperse SWCNT and observed sensitive analyte detection. An interleukin-6-binding (IL-6) aptamer, with no anchor region or spacers, was used to disperse SWCNT and directly applied for sensing (Figure 2, middle left).^[114]^ The SWCNT-aptamer construct demonstrated stability, sensitivity, and selectivity without any modification of the DNA sequence or SWCNT dispersion. Though the addition of an anchor domain was explored, the IL-6-binding aptamer alone showed superior function as a recognition probe, with an LOD of 105 ng mL^−1^. A similar approach was used to design a sensor for serotonin detection.^[115]^ A serotonin-binding aptamer was used to encapsulate SWCNT, which showed sensitive detection of the analyte with no further modification necessary. The nanosensor exhibited a physiologically relevant dynamic range of 100–1 μm. The sensor also demonstrated NIR imaging of serotonin release from platelet cells with spatiotemporal resolution. An insulin-specific aptamer was used to create a SWCNT sensor platform for detection of insulin secretion in vitro.^[116]^ The aptamer enabled sensitive and specific insulin detection with no anchor sequence or spacers. A later study using the same nanosensor proposed a framework for SWCNT detection of extracellular signaling molecules that may be broadly applied to other analytes – a biocompatible collagen extracellular matrix in which SWCNT sensors are embedded.^[111]^ These direct approaches show the efficiency and versatility of SWCNT-aptamers for biosensing.

Interestingly, SWCNT-aptamer constructs have been used to detect analytes other than those complementary to the aptamer. A hemin-binding aptamer has been used in conjunction with SWCNT transducers to create a H_2_O_2_ sensor.^[117,118]^ This sensor has been utilized to monitor H_2_O_2_ levels remotely in live plants and sense H_2_O_2_ as part of a multiplexed sensor array using different SWCNT chiralities.

Thrombin detection was achieved through noncovalent adsorption of a dye-labeled ssDNA aptamer to SWCNT, with an LOD of 1.8 nm.^[36]^ When the oligonucleotides were adsorbed to the SWCNT, an attached fluorescent dye (FAM) was brought into close proximity to the nanotube’s surface, temporarily quenching the fluorescence of FAM. The aptamer-thrombin binding event increased the distance between the dye and the SWCNT, reducing FAM quenching. This detection scheme is unique as it uses the SWCNT as a proximity quencher for a fluorescent dye. Unlike other sensors discussed here, the sensor readout is in the visible range rather than NIR. A sensor for ochratoxin A (OTA), a globally prevalent food-contaminating mycotoxin, was also created by employing a FAM-modified aptamer, with a LOD of 24.1 nm.^[119]^ Again, the SWCNT acted as a proximity quencher for FAM, which regained its fluorescence upon analyte binding.

Another unique SWCNT-aptamer platform was developed with a dual (spectrophotometry and fluorometry) readout system. The sensor detected four porphyrin species commonly found in blood: heme (FePP), protoporphyrin (PP), coproporphyrin (CP), and uroporphyrin (CP).^[120]^ SWCNT-aptamer targeting FePP was employed, which exhibits NIR fluorescence quenching upon FePP binding, exhibiting a LOD of 20 nm. Simultaneously, PP concentration was determined through the quenching of its inherent visible fluorescence upon interaction with carbon nanotubes. No changes in fluorescence were observed for CP and UP, hence, their concentrations were calculated from the Soret band absorption, once the values for FePP and PP were established.

Though noncovalent aptamer adsorption to SWCNT is more common, covalent functionalization has also been used. One study used guanine quantum defects as covalent anchors to attach ssDNA to SWCNT.^[121]^ ssDNA with a guanine-rich anchor sequence and a guanine-free capture sequence was attached to the nanotube. The previously described hemin-binding aptamer (with an added handle to hybridize with the capture sequence) along with hemin was attached as a molecular recognition unit. The sensor detected bacterial siderophores (pyoverdine and deferoxamine) through restoration of fluorescence intensity quenched by hemin. In that same manuscript, the covalent DNA anchoring strategy was used to attach a SARS CoV-2 spike protein-binding aptamer to the SWCNT. Upon recognition of the spike protein, SWCNT fluorescence decreased in a concentration-dependent manner. This versatile approach may be utilized for rational design of SWCNT sensors with high stability and specificity.

### 6.2. Complementary Nucleic Acids

One of the first SWCNT optical sensors used complementary DNA as a biorecognition element to detect hybridization events.^[38]^ The hybridization was confirmed using Forster Resonance Energy Transfer (FRET) of the DNA sequences labeled with fluorophores, with an LOD of 6 nM. SWCNT-DNA has also demonstrated utility in detection of single nucleotide polymorphisms in complementary DNA.^[122]^ Another SWCNT construct was designed to detect hybridization using a FAM-labeled hairpin DNA probe.^[123]^ The sensor exhibited high specificity toward complementary DNA and an LOD of 4 nM. The SWCNT-based sensor was compared with traditional molecular beacons in their abilities to detect DNA hybridization, finding that the SWCNT sensor offered better sensitivity and higher thermal stability.

**Figure 5.**
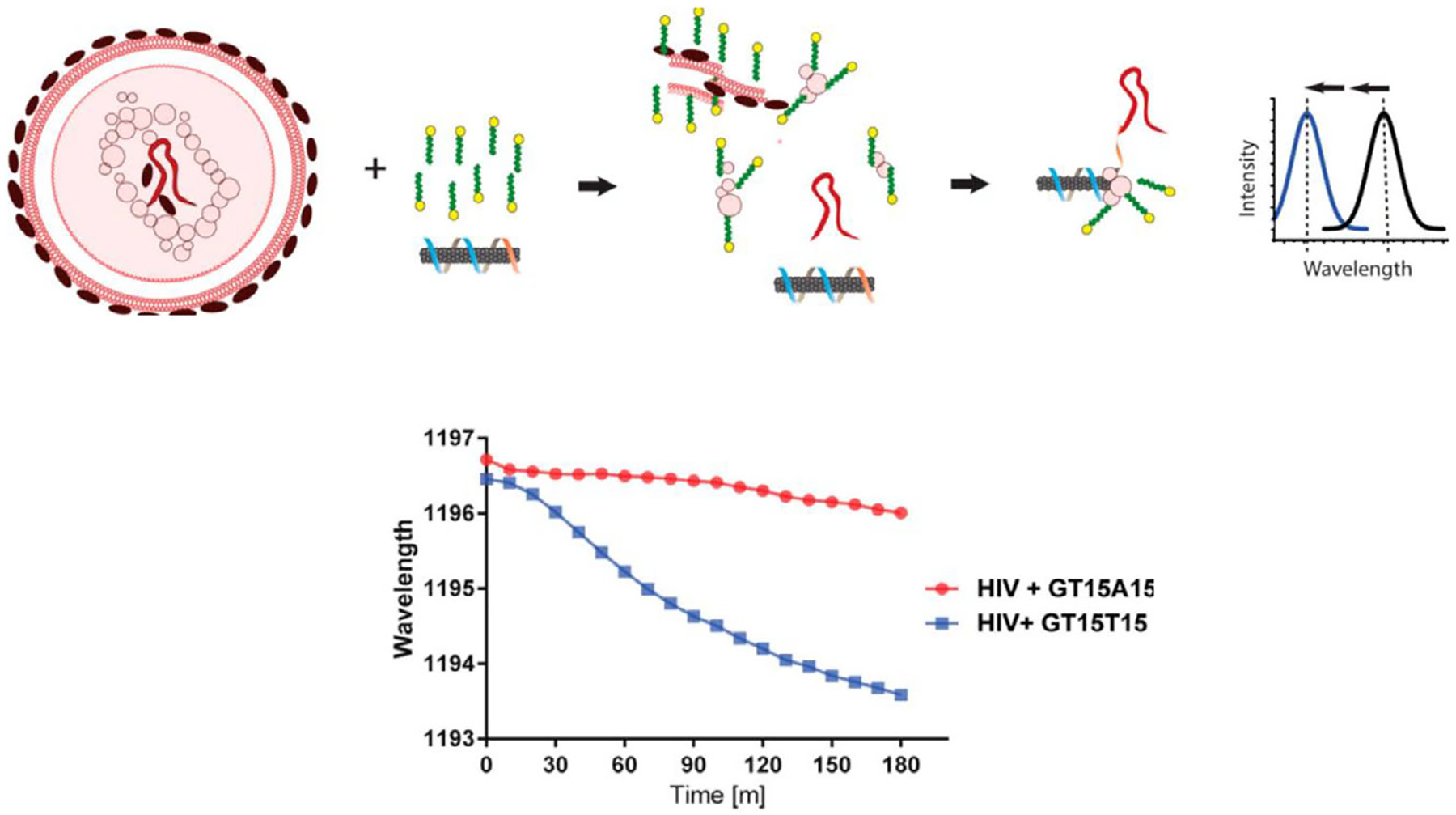
Depiction of HIV RNA detection and blue shift upon RNA hybridization with the sensor’s poly T tail. Reprinted with permission of the American Chemical Society from Harvey et al. *ACS Sensors* 2019.^[125]^

A similar SWCNT sensor performed real-time optical quantification of hybridization events of microRNA and other oligonucleotides.^[124]^ The sequence used to suspend SWCNT consisted of a DNA nanotube binding domain and a miRNA capture domain, causing a solvatochromic response when coupled with surfactants (Figure 2, middle right). The sensor was assessed in urine and serum samples as well as in vivo via an implantable device surgically inserted into the peritoneal cavity of hairless mice. The sensor detected miRNA concentrations as low as 100 pm in vivo. A similar sensor design was used to detect intact HIV in serum.^[125]^ The sensor was designed to hybridize with the polyadenylation elements of HIV RNAs. Sodium dodecyl sulfate (SDS) was used to enhance the sensor’s performance and denature the viral membrane. The denatured protein hybridized with the sensor’s poly T tail, generating a rapid and robust blue-shift in the spectrum (Figure 5).

A more recent study demonstrated a method for selecting and eliminating sensor candidates for acute myocardial infarction miRNA biomarkers. As in the previously described studies, SWCNT were suspended with a bifunctional DNA sequence containing an anchor domain and a miRNA recognition unit. The nanosensor demonstrated a fluorescence intensity increase upon binding with the target miRNA with an LOD of 10 nM. The sensor demonstrated lower response toward mutated versions of the miRNA target sequence, indicating high specificity for the target.^[126]^

### 6.3. Summary of Oligonucleotide-Based Sensors

SWCNT-DNA sensors exploit the programmability of nucleic acid sequences to produce highly tailored recognition coronas. The sensors are typically prepared through direct sonication of SWCNT and DNA, though a surfactant exchange is sometimes performed. Common strategies include aptamer integration for high-specificity targets and hybrid constructs that separate the anchor and binding domains. Optical signal transduction frequently occurs via solvatochromic shifts from corona rearrangement or intensity modulation from charge transfer during target binding. Oligonucleotides are excellent candidates for sensor recognition elements due to their chemical stability, ease of synthesis, and precise sequence control. They are, however, typically sensitive to ionic strength, pH, and nonspecific adsorption in complex samples. Successful designs often optimize base composition and secondary structure to maximize conformational change upon binding. Advances in machine learning-guided corona discovery and systematic structure-function mapping are poised to expand the scope and robustness of SWCNT-DNA sensors.

## 7. Perspective and Future Work

Optical sensing utilizing SWCNT transducers and biological recognition elements have shown great promise. The extraordinary optoelectronic properties of SWCNT and inherent specificity of biological probes synergize to create robust biosensors. These sensors have exhibited single-molecule analyte detection, high-throughput and multiplexed arrays, and quantification of relevant biomarkers in vivo.

While the library of known recognition elements is vast, there are limitations in which analytes can be targeted through biorecognition elements. Complementary antibodies or enzymes do not exist for many synthetic pharmaceuticals or small molecules. Aptamers can target some analytes that do not have complementary antibodies, but there are still many biologically relevant molecules that have not yet been the subject of aptamer development via in vitro selection. For these targets which do not have a known binding probe, alternative approaches have included the CoPhMoRe method and spectral fingerprinting techniques enabled by machine learning.^[53,127–129]^

Future work may seek to optimize site-specific, stable, and scalable functionalization strategies that preserve SWCNT optical properties while also maintaining biorecognition element activity. In addition to developing new chemistries, optimization of existing functionalization strategies (e.g. biopolymer wrappings, peptide coatings, or covalent defect chemistries) could yield substantial performance gains in applications such as drug monitoring or pollutant detection. While their clear advantage in SWCNT sensing is the known high specificity toward the desired analyte, it must be determined experimentally if the recognition event will induce a meaningful modulation in SWCNT fluorescence. In some instances, attachment of the recognition element to SWCNT alters its conformation and therefore its analyte binding activity.

Various studies have sought to understand the mechanism of fluorescence modulation in SWCNT sensing.^[60,130]^ This commonly occurs through conformational change of the recognition element or dispersing agent (such as ssDNA) or bringing the analyte close to the surface of the SWCNT which induced a charge transfer or modifies the local dielectric environment. To accelerate sensor design, systematic variations of functional groups and corona chemistries could be used to map structure-function correlations, linking molecular interactions to optical modulation mechanisms. Greater understanding of fluorescence mechanisms will lead to more efficient sensor design.

Enhancement of sensitivity and selectivity may also be explored. Engineering of biorecognition elements with higher affinity and specificity, as well as utilization of individual SWCNT chiralities for ratiometric/multiplexed sensing platforms, may lead to ultrasensitive devices with unprecedented abilities. Beyond proof-of-concept demonstrations, deploying these systems as spatiotemporal tracking platforms to monitor targets in physiologically or environmentally relevant conditions would provide critical insight into their real-world performance. Although some of the SWCNT sensors discussed here were deployed in vivo, further research into long-term sensor performance is still needed to ensure continuous monitoring of biomarkers for translation to the clinic. Given the promise and potential, the combination of biologically active recognition elements with powerful SWCNT optical transducers offers a unique capacity for clinically relevant diagnostic tool development.

## Figures and Tables

**Table 1.** Protein-Based SWCNT Optical Sensors.

Analyte	Biorecognition Element	Limit of Detection	Signal Transduction	Clinical Relevance	Reference
Cardiac troponin T (cTnT)	Anti-cTnT antibody	100 ng mL^−1^ in buffer 7 μg mL^−1^ in plasma	Fluorescence intensity increase	Healthy range: 0–0.01 ng mL^−1^	[70]
Urokinase plasminogen activator (uPA)	Anti-uPA antibody	100 pm in buffer 25 nm in serum	Red-shift	Disease range: up to 17 nm	[41]
Human epididymis protein 4 (HE4)	Anti-HE4 antibody	10 nm in serum	Blue-shift	Disease range: up to 10 nm	[42]
Interleukin-6 (IL-6)	Anti-IL-6 antibody	25 pg mL^−1^ in serum	Red-shift	Disease range: 0.005 – 500 ng mL^−1^	[71]
Estrogen receptor alpha (ERa)	Anti-ERa antibody	Not reported	Red-shift	Binary cell surface expression	[44]
Rituxan, Herceptin	CD20, HER2/neu	Not reported	NIR fluorescence	Binary cell surface expression	[72]
Prostate-specific membrane antigen (PSMA)	Anti-PSMA antibody	Not reported	NIR fluorescence	Binary cell surface expression	[73,74]
*S. aureus*	Anti- *S. aureus* antibody	Not reported	NIR fluorescence	Whole-organism	[75]
Green fluorescent protein (GFP)	GFP-binding nanobody	Not reported	NIR fluorescence / GFP fluorescence	Optical-based detection	[46,78]
Interleukin-6 (IL-6)	Anti-IL-6 supercharged antibody fragment	80 nm	Red-shift	Disease range: 0.005 – 500 ng mL^−1^	[79]
Glucose	Glucose oxidase	34.7 μm ^[10,11]^ 42 μm ^[84]^	Fluorescence intensity increase	Healthy range: > 100 mg dL^−1^ Disease range: 126 mg dL^−1^ or above	[10,11,80,81,84,98]
ATP	Luciferase	240 nm	Fluorescence intensity quenching	Optical-based detection	[85]
SARS-CoV-2 spike protein	Angiotensin converting enzyme 2	≈10^4^ viral copies per μL	Fluorescence intensity increase	≈10–10^4^ viral copies per μL	[86]
Hydrogen peroxide	Horseradish peroxidase	31 μm	Fluorescence intensity increase	Healthy range: 1–5 μm	[87]
p16, CDk4, FOS, JUN, IgG, IgM, IgG2a, IgD, glycans	His-tagged capture proteins	10 ng mL^−1^ IgG ^[92]^ 25 μg mL^−1^ IgG ^[93]^	Fluorescence intensity increase	Protein tags rather than disease biomarkers	[19,90–93]
Avidin	Biotin	Not reported	Fluorescence intensity increase	Affinity capture rather than quantitation	[94]
Glucose	Glucose binding protein	2.5 mm	Fluorescence intensity quenching	Healthy range: > 100 mg dL^−1^ Disease range: 126 mg dL^−1^ or above	[95]
Glucose	Concanavalin A	Not reported	Fluorescence intensity increase	Healthy range: > 100 mg dL^−1^ Disease range: 126 mg dL^−1^ or above	[96]
Kinesin-1-motor protein	HaloTag	Not reported	Near-IR fluorescence	Intracellular protein	[97]

**Table 2.** Peptide-Based SWCNT Optical Sensors.

Analyte	Biorecognition Element	Limit of Detection	Signal Transduction	Clinical Relevance	Reference
Nitroaromatics	Bombolitin II	Not reported	Fluorescence intensity quenching (ratiometric)	N/A	[104]
Integrins	Integrin-binding peptide sequence (Arg-Gly-As)	Not reported	NIR fluorescence	Intracellular rather than in solution	[105]
Amyloid-beta (A*β*)	A*β*_42_ peptide	100 nm in buffer	Fluorescence intensity increase and blue-shift	Disease range: 300 – 600 pg mL^−1^	[106]
Various bacterial lipopolysaccharides	Lipopolysaccharide (LPS)-binding peptide	Not reported	Fluorescence intensity increase	Part of the bacterial well wall	[107]
Glutathione s-transferase (GST) proteins and GST-fusion proteins	Tripeptide glutathione	1 nm	Fluorescence increase and red-shift	Protein tag rather than free full protein	[108]

**Table 3.** Oligonucleotide-Based SWCNT Optical Sensors.

Analyte	Biorecognition Element	Limit of Detection	Signal Transduction	Clinical Relevance	Reference
RAP1, HIV1 integrase	RAP1-binding aptamer, HIV1 integrase-binding aptamer	Not reported	Fluorescence intensity increase	Not reported	[39]
Insulin	Insulin-binding aptamer	0.13 μg mL^−1^	Fluorescence intensity quenching	< 174 pm fasting, up to 1150 pm after eating	[112]
Platelet-derived growth factor (PDGF)	PDGF-binding aptamer	0.1 nm	Fluorescence intensity quenching	Healthy range: 128 – 208 pg mL^−1^	[113]
Interleukin-6 (IL-6)	IL-6-binding aptamer	105 ng mL^−1^	Fluorescence intensity quenching	Disease range: 0.005 – 500 ng mL ^−1^	[114]
Serotonin	Serotonin-binding aptamer	100 nm	Fluorescence intensity increase	Healthy range: 100 – 300 ng mL^−1^	[115]
H_2_O_2_	Hemin-binding aptamer + hemin	10μm	Fluorescence intensity quenching	10 – 100 μm in plants	[117,118]
Thrombin	FAM-labeled thrombin-binding aptamer	1.8 nm	FAM fluorescence intensity increase	3–32 pm	[36]
Ochratoxin A (OTA)	FAM-labeled OTA-binding aptamer	24.1 nm	FAM fluorescence intensity increase	Not reported	[119]
Heme	Heme-binding aptamer	20 nm	Fluorescence intensity quenching	Disease range: 35 – 150 μM	[120]
SARS CoV-2 spike protein	Covalent DNA anchor + SARS CoV-2 spike protein-binding aptamer	0.75 nm	Fluorescence intensity quenching	33.9 ± 22.4 pg mL^−1^	[121]
Complementary DNA sequence	24-mer ssDNA sequence	6 nm	Blue-shift	Intracellular/genomic, rather than disease-specific	[38]
Single-nucleotide polymorphism	24-mer ssDNA sequence	Not reported	Red-shift	Intracellular/genomic rather than soluble	[122]
Complementary DNA sequence	FAM-labeled 31-mer ssDNA sequence	4 nm	FAM fluorescence intensity increase	Intracellular/genomic rather than soluble	[123]
Complementary miRNA sequence	(GT)_15_ ssDNA + miRNA capture sequence	10 – 100 pm	Blue-shift	Intracellular/genomic rather than soluble	[124]
Polyadenylation elements of HIV RNAs	(GT)_15_-(T)_15_ ssDNA	Not reported	Blue-shift	Intracellular/genomic rather than soluble	[125]
Myocardial infarction miRNA biomarkers	(GT)_15_ ssDNA + miRNA capture sequence	10 nm	Fluorescence intensity increase	10 fm - 100 pm	[126]
